# Astemizole Synergizes Calcitriol Antiproliferative Activity by Inhibiting CYP24A1 and Upregulating VDR: A Novel Approach for Breast Cancer Therapy

**DOI:** 10.1371/journal.pone.0045063

**Published:** 2012-09-12

**Authors:** Janice García-Quiroz, Rocío García-Becerra, David Barrera, Nancy Santos, Euclides Avila, David Ordaz-Rosado, Mariana Rivas-Suárez, Ali Halhali, Pamela Rodríguez, Armando Gamboa-Domínguez, Heriberto Medina-Franco, Javier Camacho, Fernando Larrea, Lorenza Díaz

**Affiliations:** 1 Departamento de Biología de la Reproducción, Instituto Nacional de Ciencias Médicas y Nutrición Salvador Zubirán, México, D.F., México; 2 Departamento de Farmacología, Centro de Investigación y de Estudios Avanzados del I.P.N., México, D.F., México; 3 Departamento de Cirugía, Instituto Nacional de Ciencias Médicas y Nutrición Salvador Zubirán, México, D.F., México; 4 Departamento de Patología, Instituto Nacional de Ciencias Médicas y Nutrición Salvador Zubirán, México, D.F., México; Roswell Park Cancer Institute, United States of America

## Abstract

**Background:**

Calcitriol antiproliferative effects include inhibition of the oncogenic *ether-à-go-go-1* potassium channel (Eag1) expression, which is necessary for cell cycle progression and tumorigenesis. Astemizole, a new promising antineoplastic drug, targets Eag1 by blocking ion currents. Herein, we characterized the interaction between calcitriol and astemizole as well as their conjoint antiproliferative action in SUM-229PE, T-47D and primary tumor-derived breast cancer cells.

**Methodology/Principal Findings:**

Molecular markers were studied by immunocytochemistry, Western blot and real time PCR. Inhibitory concentrations were determined by dose-response curves and metabolic activity assays. At clinically achievable drug concentrations, synergistic antiproliferative interaction was observed between calcitriol and astemizole, as calculated by combination index analysis (CI <1). Astemizole significantly enhanced calcitriol’s growth-inhibitory effects (3–11 folds, *P*<0.01). Mean IC_20_ values were 1.82±2.41 nM and 1.62±0.75 µM; for calcitriol (in estrogen receptor negative cells) and astemizole, respectively. Real time PCR showed that both drugs alone downregulated, while simultaneous treatment further reduced Ki-67 and Eag1 gene expression (*P*<0.05). Astemizole inhibited basal and calcitriol-induced CYP24A1 and CYP3A4 mRNA expression (cytochromes involved in calcitriol and astemizole degradation) in breast and hepatoma cancer cells, respectively, while upregulated vitamin D receptor (VDR) expression.

**Conclusions/Significance:**

Astemizole synergized calcitriol antiproliferative effects by downregulating CYP24A1, upregulating VDR and targeting Eag1. This study provides insight into the molecular mechanisms involved in astemizole-calcitriol combined antineoplastic effect, offering scientific support to test both compounds in combination in further preclinical and clinical studies of neoplasms expressing VDR and Eag1. VDR-negative tumors might also be sensitized to calcitriol antineoplastic effects by the use of astemizole. Herein we suggest a novel combined adjuvant therapy for the management of VDR/Eag1-expressing breast cancer tumors. Since astemizole improves calcitriol bioavailability and activity, decreased calcitriol dosing is advised for conjoint administration.

## Introduction

Regulation of vitamin D metabolism is pivotal for human physiology, since the hormonal form calcitriol, acting through the vitamin D receptor (VDR), controls gene expression programs mainly associated with calcium homeostasis. Moreover, calcitriol is an important endogenous anti-cancer agent due to its ability to modulate master regulatory networks resulting in the inhibition of cell proliferation, acquisition of a more differentiated phenotype and induction of apoptosis [1]–[3]. Among the various mechanisms involved in the antineoplastic effects of calcitriol, downregulation of the oncogenic *ether-à-go-go-1* potassium channel (Eag1, Kv10.1, KCNH1) expression plays a central role for cell growth inhibition [4], [5].

Breast cancer is the most frequently diagnosed neoplasia and leading cause of cancer death among women [6]. Epidemiologic studies have shown that low serum vitamin D levels correlate with increased risk of breast cancer, disease progression and bone metastases; while disruption of VDR signaling in the breast gland is associated with higher incidence of preneoplastic lesions [7]–[9]. Furthermore, over 90% of human breast cancers express the VDR, which correlates with a longer disease-free interval compared to patients with VDR-negative tumors [10]. Besides the presence of a functional VDR, an important consideration for successful calcitriol-based anticancer treatment is the level of vitamin D 24-hydroxylase (CYP24A1), which is the enzyme that degrades calcitriol. In normal cells, basal CYP24A1 expression is generally undetectable; however, it is usually overexpressed in several malignancies, suggesting its involvement in tumorigenesis [11]. Therefore, specific drugs able to suppress calcitriol catabolism by targeting CYP24A1 constitute an attractive strategy to potentiate its antitumoral activity. As an autoregulatory mechanism, the expression of CYP24A1 is under direct regulation of calcitriol through its binding to VDR and further interaction with vitamin D response elements (VDRE) that exist in CYP24A1 promoter.

New approaches towards fighting breast cancer include combined targeted systemic adjuvant therapies appropriate for heterogeneous tumors expressing different sets of molecular signatures. Previously, we showed that co-incubation of calcitriol together with astemizole, a nonspecific Eag1 inhibitor, reduced breast cancer cell proliferation to a greater extent than using either drug alone [4]. Eag1 promotes oncogenesis, proliferation and tumor progression; and therefore it is used as a marker and therapeutic target for several types of cancers [12], [13]. Indeed, Eag1 shows restricted distribution in healthy tissues such as the central nervous system, but otherwise is abundantly expressed in malignant cell lines and primary tumors [14], [15]. Particularly in breast cancer, several studies have established that Eag1 K^+^ channels are crucial for proliferation, cell cycle progression and vascularization [16], [17]. Therefore, the rationale of the combined therapy proposed herein is based on Eag1 gene expression inhibition by calcitriol, together with the functional blockade of K^+^ currents through this particular channel by astemizole, in order to potentiate the antineoplastic effects of both compounds. Astemizole, used for many years as an H_1_-histamine receptor antagonist, is a long-acting, non-sedating second-generation anti-histamine currently used in some countries to treat allergy symptoms. However, astemizole has recently gained interest as an antineoplastic drug since it targets important ion channels involved in cancer progression, such as Eag1 [18]. Astemizole permeates the cell membrane and inhibits Eag1 currents by selectively binding to open channels. It does not significantly infiltrate the blood brain barrier, and therefore does not cause depression of the central nervous system. As in the case of calcitriol, astemizole antineoplastic effects involve different mechanisms of action which may further improve their conjoint therapeutic action, such as antagonizing H_1_-histamine receptors [18], reducing P450-aromatase expression [19] and inhibiting the release of inflammatory mediators [20]. Thus, acting through different pathways both compounds have proven to promote cell cycle arrest and apoptosis while inhibit tumor progression *in vivo* and *in vitro* [1], [18]. As a reference, physiologic levels of calcitriol range from 0.05 to 0.16 nM, while clinical studies have demonstrated that under a weekly administration schedule, calcitriol may reach peak blood levels of 3–16 nM with little toxicity [21], [22]. In the case of astemizole, reported therapeutic and toxic serum levels are 0.05 µg/mL (0.10 µM) and 14 µg/mL (30.5 µM), respectively [23].

Therefore, in the present study, we expand our previous work by further investigating the interaction between calcitriol and astemizol in order to determine the nature of their combined action and reciprocal effects on bioavailability, with the aim to establish scientific criteria for a combined adjuvant therapy applicable to different kinds of breast tumors independently of estrogen or growth factor-receptors status. Indeed, by taking advantage of VDR and Eag1 increased expression in a high number of breast cancer tumors [4], [15], [24], this therapeutic approach could be validated in future preclinical studies and clinical trials of mammary carcinoma or any other malignant neoplasm over-expressing Eag1 and VDR.

## Methods

### Reagents

Culture media, fetal bovine serum (FBS), Trizol and the oligonucleotides for real time polymerase chain reaction (PCR) were from Invitrogen (CA, USA). The TaqMan Master reaction, TaqMan probes, capillaries, probes, reverse transcription (RT) system and the cell proliferation assay (XTT) were from Roche (Roche Applied Science, IN, USA). Calcitriol (1,25-dihydroxycholecalciferol) and astemizole were kindly donated from Hoffmann-La Roche Ltd (Basel, Switzerland) and Liomont (México City, México), respectively. 3,3′-diaminobenzidine tetrahydrochloride substrate kit (DAB) was from BioSB (CA, USA).

### Ethics Statement

Samples from breast cancer were obtained at the Instituto Nacional de Ciencias Médicas y Nutrición Salvador Zubirán in México City. The protocol was approved by the Institutional Human Research Ethics Committee (Comité Institucional de Investigación Biomédica en Humanos, CIIBH) and written informed consent was obtained from donors.

### Human Tissues

Biopsies were harvested during lumpectomy from two female patients diagnosed with invasive ductal carcinoma (IDC). Two established human cell lines isolated from pleural effusions of IDC were also used in this study: SUM-229PE (Asterand, San Francisco, CA) and T-47D (ATCC, Manassas, VA). The human hepatocellular carcinoma cell line HuH-7 (Japanese Collection of Resesarch Bioresources), utilized to study CYP3A4 regulation, was kindly donated by Dr. María Rivas (Universidad Autónoma de Nuevo Leon, Mexico).

### Cell Culture

Primary cell cultures were derived from fresh human IDC biopsies as described previously [4], [25]. Cells were allowed to grow out from cultured explants in supplemented DMEM-F12 medium (100 U/ml penicillin, 100 µg/ml streptomycin), containing 5% heat-inactivated-FBS. Incubations were performed in humidified 5% CO2-95% air at 37°C. Once 70% confluence was reached, subculturing was performed by tripsinization (trypsin/EDTA 0.25%/0.2 g/L). After approximately 5 passages, tumor-derived cells were characterized by immunocytochemistry and were further referred as IDC-1 and IDC-2. Commercial cell lines were maintained following indications from suppliers. All experimental procedures were performed in supplemented DMEM-F12 medium conditioned with 5% charcoal-stripped-heat-inactivated FBS.

### Immunocytochemistry

Cultured cells were grown on glass coverslips and fixated in ethanol 96%. Antigen retrieval was done by autoclaving in EDTA (0.1 M, pH 9.0). Slides were blocked with immunodetector peroxidase blocker (BioSB). For Eag1 additional blocking was performed using background Sniper (Biocare Medical, CA, USA). The following primary antibodies were incubated in order to immunocharacterize cells: estrogen receptor (ERα) and VDR (1∶100, Santa Cruz Biotechnology Inc, CA, USA), epidermal growth factor receptor-2 (Her-2/neu, 1∶100, Dako, Glostrup, Denmark), progesterone receptor (PR, 1∶100, BioSB) and Eag1 (1∶300, Novus Biologicals CO, USA). After washing, the slides were sequentially incubated with immune-Detector Biotin-Link and Immuno-Detector HRP label (Bio SB) during 10 minutes each. Staining was completed with DAB.

In order to analyze the effect of the drugs upon Ki-67 at the protein level, cells grown on glass coverslips were cultured in the presence of calcitriol, astemizole or both (IC_20_ in all cases) or else vehicle during 48 h.

Afterwards cells were fixated and processed as described above using a monoclonal anti-Ki-67. No hematoxylin counterstaining was performed. The percentage of Ki-67 positivity was calculated by counting total Ki67-positive nuclei × 100/total cell count. SUM-229PE, T-47D and IDC-2 were analyzed, total cells were counted in at least 3 different fields per treatment and 4 different observers participated in the analysis.

### Proliferation and Drug Combination Studies

Cells were plated in 96-well microdilution plates. In each well a constant amount of cells was seeded (200–1000 depending on the cell line). After 24 h medium containing vehicle, astemizole, calcitriol or the combination of both was added in sextuplicate and incubated during 6 days. Afterwards, cell proliferation was measured by using the colorimetric XTT assay following manufacturer’s instructions. Absorbance in each well was determined at 492 nm in a Multiskan spectrophotometer (Labsystems Inc, Canada). Inhibitory concentration (IC) values were calculated by non-linear regression analysis using sigmoidal fitting from the sigmoidal dose-response curve by means of the scientific graphing software Origin (OriginLab Corporation, Northampton, MA). The IC_50_ is defined as the concentration of the compound that caused 50% inhibition of cell proliferation within the range of concentrations tested (calcitriol: 0.1 nM–1 µM, astemizole: 0.5–4.5 µM). Growth inhibitory effects by the corresponding IC_20_ in each cell line were calculated as percentage inhibition as follows: absorbance of treated cells × 100/absorbance of vehicle-treated cells. The resulting value was then subtracted from 100 to obtain final percent growth inhibition.

Combination index values (CI) were derived from sextuplicate dose-response curves applying the multiple drug-effect equation of Chou-Talalay [26]. For this analysis synergy is defined as CI values <1.0, antagonism as CI values >1.0, and additivity as CI values = 1.0.

### Real Time PCR

Cells were incubated in the presence of different calcitriol and/or astemizole concentrations or their respective vehicle (0.1% ethanol/DMSO) during 24 hours. Afterwards, medium was aspirated and RNA extracted using Trizol reagent. Three µg of total RNA were reverse transcribed with the transcriptor RT system. Real-time PCR was carried out using the LightCycler® 2.0 from Roche (Roche Diagnostics, Mannheim, Germany), according to the following protocol: activation of Taq DNA polymerase and DNA denaturation at 95°C for 10 min, proceeded by 45 amplification cycles consisting of 10 s at 95°C, 30 s at 60°C, and 1 s at 72°C. Primers sequences, corresponding probe numbers and sizes of resulting amplicons are given in Table 1. Gene expression of the housekeeping gene glyceraldehyde-3-phosphate dehydrogenase (GAPDH) was used as an internal control, while Ki-67 was used as a molecular marker for cell proliferation.

**Table 1:** Primers and probes.

Gen/Accession number	Upper primer	Lower primer	Amplicon (nt)	Probe number*
Eag1/AF078741.1	cct gga ggt gat cca aga tg	cca aac acg tct cct ttt cc	60	49
Ki-67/X65550.1	ggt gtg cag aaa atc caa aga	act gtc cct atg act tct ggt tg	77	73
CYP24A1/NM_000782.3	cat cat ggc cat caa aac aa	gca gct cga ctg gag tga c	65	88
CYP3A4/NM_017460.3	gat ggc tct cat ccc aga ctt	agt cca tgt gaa tgg gtt cc	96	2
VDR/NM_000376.1	gtg aga cct cac aga aga gca c	cat tgc ctc cat ccc tga	72	68
GAPDH/AF261085.1	agc cac atc gct gag aca c	gcc caa tac gac caa atc c	66	60

* From the universal probe library (Roche).

### Western Blot

Cells were treated with calcitriol and/or astemizole (IC_20_) or vehicle during 48 hours. Afterwards, medium was aspirated and cells were trypsinized, pelleted and lysed with RIPA buffer (9.1 mM dibasic sodium phosphate, 1.7 mM monobasic sodium phosphate, 150 mM NaCl, 1% Nonidet P-40, 0.1% SDS, pH 7.4) in the presence of a protease inhibitor cocktail. Total cell lysates (50 µg) were separated on 10% SDS-PAGE, transferred to PVDF membranes, and blocked with 10% skim milk. Membranes were incubated with anti-VDR antibody (1∶200, C-20 sc1008, Santa Cruz Biotechnology Inc, CA, USA) during 24 h at 4°C. For visualization, membranes were incubated with a horseradish peroxidase-conjugated secondary antibody (1∶1000) and were processed with the ECL detection system (Amersham Pharmacia UK). For normalization, blots were stripped in boiling stripping buffer (2% w/v SDS, 62.5 mM Tris-HCl pH 6.8, 100 mM 2-mercapto-ethanol) for 10 minutes and sequentially incubated with anti-GAPDH (1∶10000, Millipore, USA) and anti-mouse-HRP (1∶10000, Jackson ImmunoResearch Laboratories, Inc).

### Statistical Analysis

Data are expressed as the mean ± standard deviation (S.D.). Statistical differences for dose-response assays were determined by One-Way ANOVA followed by appropriate post-hoc test (Holm-Sidak method for pair-wise comparisons), using a specialized software package (SigmaStat, Jandel Scientific). Differences were considered statistically significant at *P*<0.05.

## Results

### Cell Characterization

The cell lines T-47D, SUM-229PE and two primary IDC-derived (IDC-1 and IDC-2) were representative of different breast cancer subtypes, based on the expression of PR, ERα and Her2-neu. All cell lines were positive for VDR and Eag1, whereas three out of four were ER-negative (Table 2).

**Table 2:** Cell characterization by immunocytochemistry.

Cell line	ERα	PR	Her2-neu	Eag1	VDR
SUM-229 PE	–	–	+	+	+
T-47D	+	+	–	+	+
IDC-1	–	–	–	+	+
IDC-2	–	–	+	+	+

### Antiproliferative Effects of Calcitriol and Astemizole

**Figure 1:**
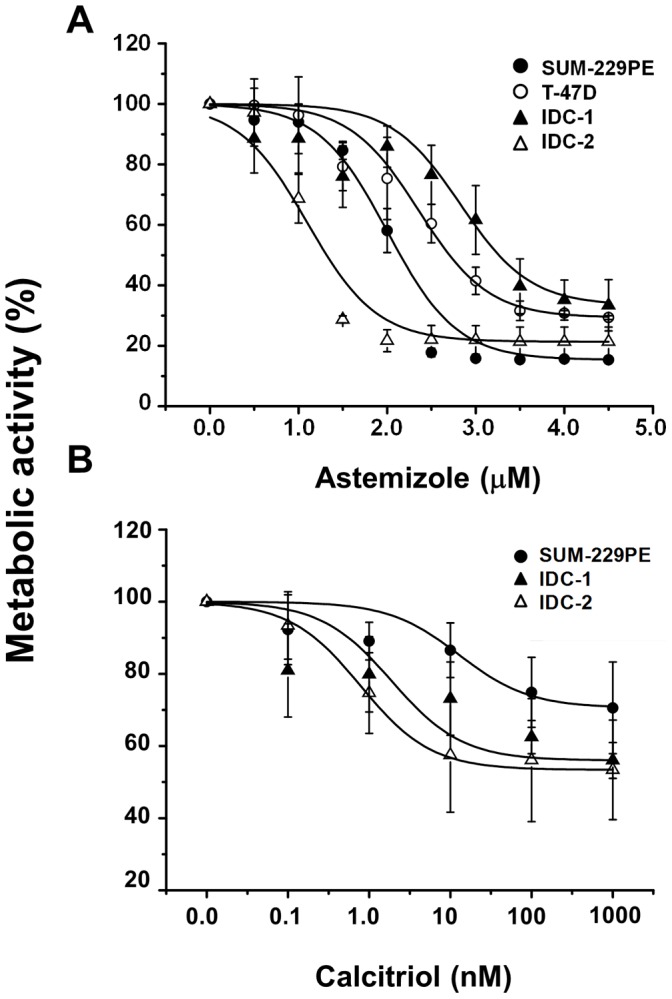
Calcitriol and astemizole antiproliferative effects in cultured breast cancer cells. Cells were incubated in the presence of different astemizole (A) or calcitriol (B) concentrations during 6 days. The culture media was not changed during the incubation period. Proliferation was determined measuring the metabolic activity of viable cells by using the XTT colorimetric method. Both calcitriol and astemizole inhibited cell proliferation in a concentration-dependent manner. Results are the mean ± S.D. of sextuplicate determinations and represent at least three different experiments. Based on the IC_50_ values, the sensitivity to astemizole was: IDC-2> SUM-229PE > T-47D > IDC-1, while for calcitriol was: IDC-2> IDC-1> SUM-229PE. T-47D was rather resistant to calcitriol and therefore omitted from the graph. One-way ANOVA followed by Dunnett's test indicated a significant statistical significance (*P*<0.001) for each concentration tested vs vehicle, starting from 1.5 µM (A) and 1 nM (B). The only exception was IDC-2 in panel (A), which was significant (*P*<0.001) starting from 1.0 µM.

Concentration-dependent antiproliferative effects of calcitriol and astemizole were observed in all cells, but varied significantly between individual cell lines (Figure 1A and 1B). Based on the calculated IC_50_ values (Table 3), the sensitivity to astemizole was: IDC-2> SUM-229PE > T-47D > IDC-1, while for calcitriol was: IDC-2> IDC-1> SUM-229PE > T-47D. T-47D was rather resistant to calcitriol; therefore, the IC_50_ was calculated in the range between 0.1 nM–10 µM in these cells.

**Table 3:** IC values for astemizole and calcitriol.

	Astemizole		Calcitriol	
Cell line	IC_20_ (µM)	IC_50_ (µM)	IC_20_ (nM)	IC_50_ (nM)
SUM-229 PE	1.76	2.02	4.60	31.00
T-47D	1.30	2.70	858.0	2630.00
IDC-1	2.60	3.01	0.57	8.00
IDC-2	0.83	1.08	0.28	0.96
Mean ± S.D.	1.62±0.75	2.20±0.85	1.82±2.41^a^	13.32±15.71^a^

a Mean values for calcitriol were calculated without considering T-47D.

### Effects of the Combined Treatment of Calcitriol and Astemizole upon Cell Proliferation

We next analyzed the combination of calcitriol plus astemizole on breast cancer cell proliferation. Table 4 shows the growth inhibitory effects achieved in SUM-229PE, T-47D and IDC-1when incubating the cells in the presence of calcitriol and astemizole alone or in combination at corresponding IC_20_ values. As depicted, percent growth inhibition was significantly improved when both drugs were coincubated.

**Table 4:** Growth inhibitory effects (%) exerted by astemizole and calcitriol alone or in combination (C + A) using IC_20_ values.

Cell line	Astemizole	Calcitriol	C + A
SUM-229PE	13.55±0.964	4.67±4.34	53.27±4.34*
T-47D	9.97±2.15	7.67±0.07	51.26±8.52*
IDC-1	41.9±6.99	20.91±2.77	65.63±5.15*

Results are expressed as the mean ± S.D. percent growth inhibition. *P<0.05 vs. each drug alone, n = 6.

Since IDC-2 was highly sensitive to calcitriol, we decided to test it at concentrations below the IC_20_ in combination with a therapeutic concentration of astemizole in this cell line and SUM-229PE. As depicted in Table 5, astemizole (1 µM) or calcitriol (0.1 nM) alone slightly inhibited cell growth in SUM-229PE; but when used concomitantly, a significant inhibition of cell proliferation was observed. Similar results were obtained in IDC-2 using a much lower calcitriol dose (0.01 nM, Table 5).

**Table 5:** Growth inhibitory effects (%) exerted by astemizole and calcitriol alone or in combination (C + A) using drug concentrations below the IC_20_ values.

Cell line	Astemizole	Calcitriol	C + A
SUM-229PE	3.26±0.273	1.94±6.31	34.87±4.43*
IDC-2	49.60±6.98	10.17±0.396	59.09±5.27*

Results are expressed as the mean ± S.D. percent growth inhibition. *P<0.05 vs. each drug alone, n = 6. Concentrations used were: Astemizole: 1 µM; Calcitriol: 0.1 nM and 0.01 nM for SUM-229PE and IDC-2, respectively.

In order to quantitatively determine the nature of calcitriol and astemizole interaction upon cell proliferation, multiple drug effect analysis was performed using the IC_50_ values of each compound. As shown in Table 6, CI values ranged from 0.67 to 0.96, depicting synergistic interactions for the combination of both drugs in all cell lines tested.

**Table 6:** CI values.

	CI	Outcome
SUM-229 PE	0.676	Synergy
T-47D	0.784	Synergy
IDC-1	0.693	Synergy
IDC-2	0.964	Synergy

CI values were calculated using the combination index (CI) equation. CI <1, = 1, and >1 indicate synergic, additive, or antagonic effects; respectively.

### Evaluation of the Molecular Markers Eag1 and Ki-67 in Response to Co-treatment

For the drugs under investigation we studied their ability to conjointly inhibit proliferation as a biologic endpoint. However; as an additional clinically relevant parameter, we also studied the effect of calcitriol and astemizole upon the gene expression of their shared molecular target Eag1, as well as upon the proliferation indicator Ki-67. For these studies we used IDC-1 and SUM-229PE cell lines.

**Figure 2:**
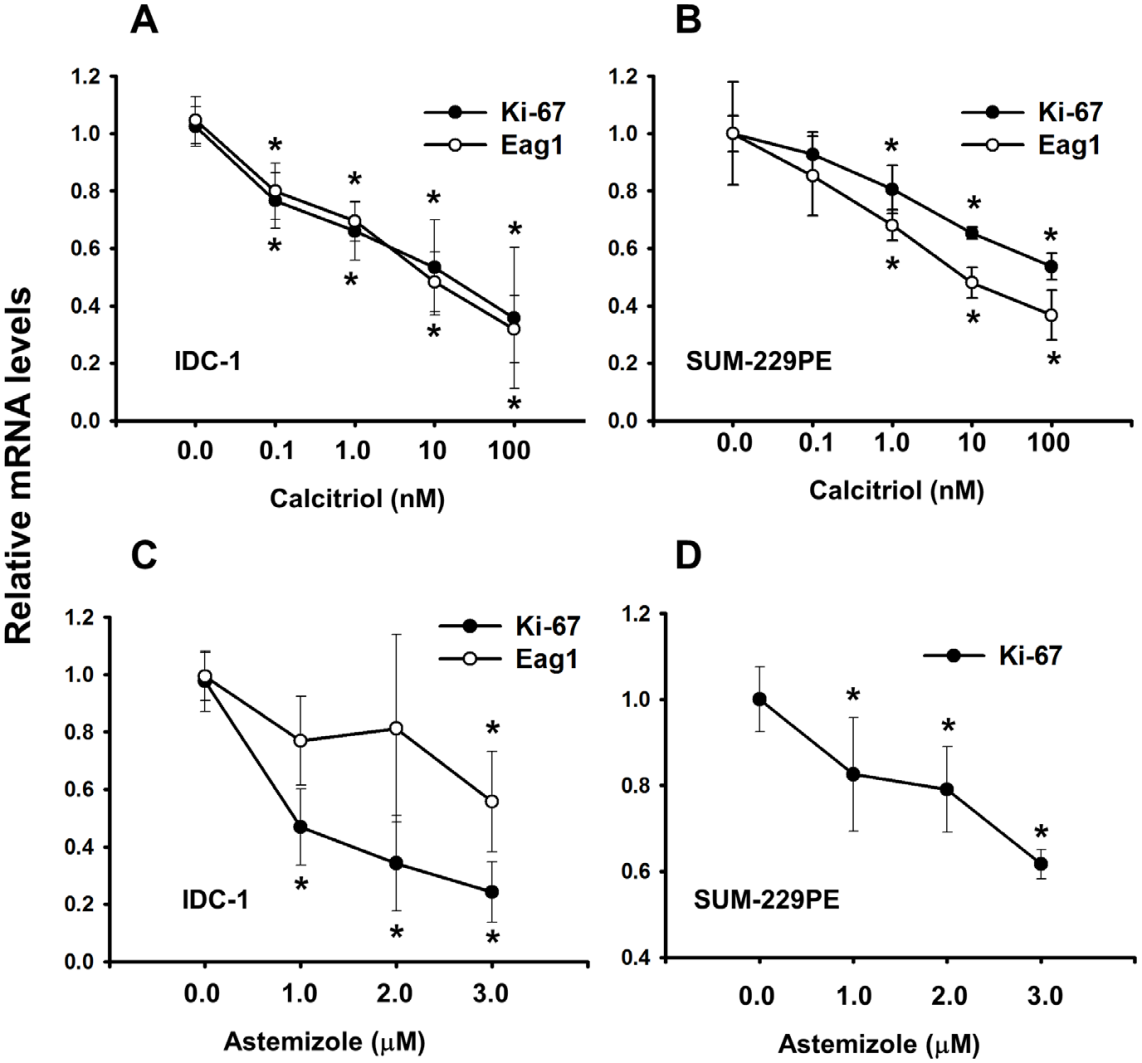
Ki-67 and Eag1 are transcriptionally downregulated by calcitriol and astemizole in breast cancer cells. The gene expression of Ki-67 (dark circle) and Eag1 (white circle) was downregulated by calcitriol (A–B) and astemizole (C–D) in IDC-1 (A, C) and SUM-229PE cells (B, D). Eag1 was not regulated by astemizole in SUM-229PE (data not shown). Relative Ki-67 and Eag1 mRNA levels were obtained by normalizing against GAPDH mRNA expression. Vehicle values were set to one. N = at least 3, *P<0.05 vs control.

Calcitriol dose-responsively inhibited Eag1 and Ki-67 gene expression in both cell lines (*P*<0.05, Figure 2A and 2B). Likewise, astemizole significantly inhibited Ki-67 gene expression in a concentration-dependent manner (Fig. 2C and 2D). A similar tendency was observed with the regulation of Eag1 by astemizole in IDC-1, but statistical significance was reached only at a high concentration (Fig. 2C) and without effect in SUM-229PE (data not shown).

**Figure 3:**
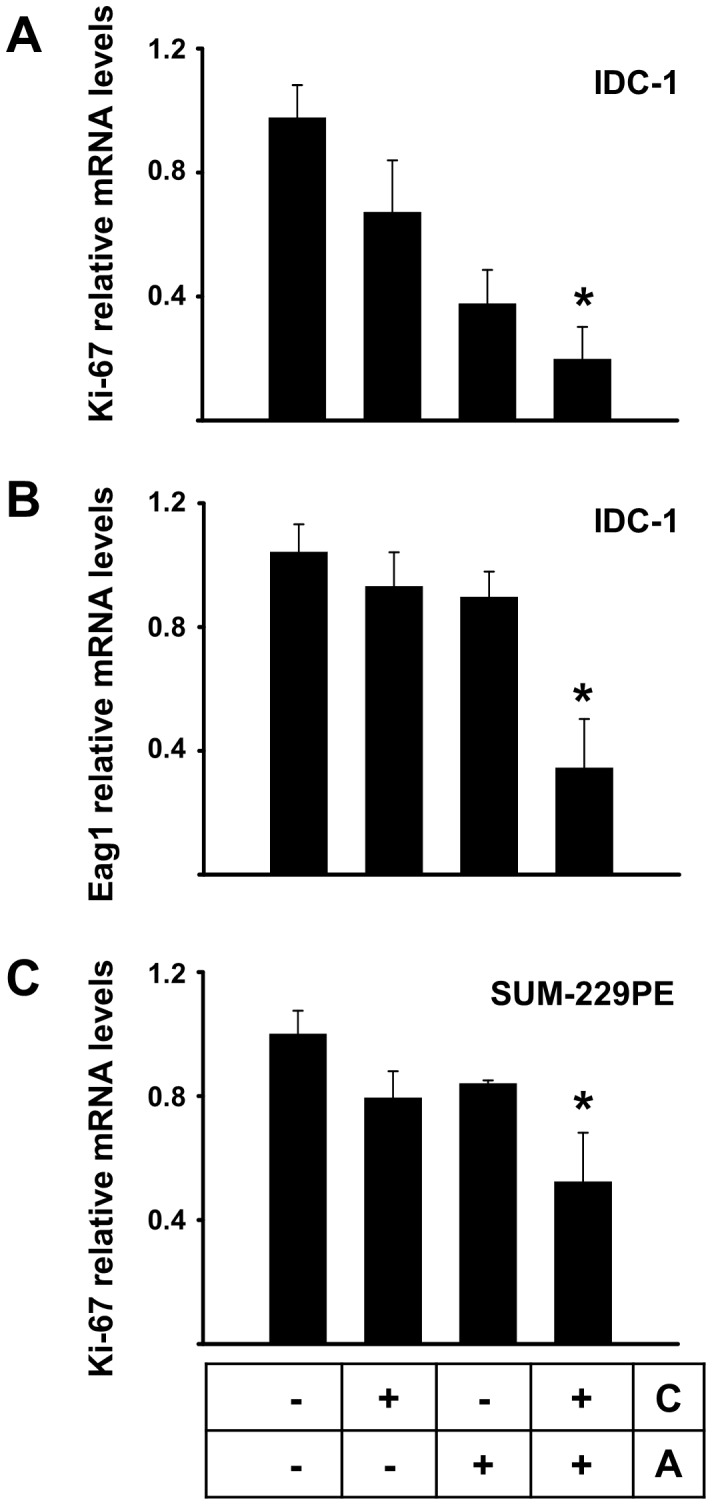
Calcitriol and astemizole synergistically downregulate mRNA expression of the molecular markers Ki-67 and Eag1. Two calcitriol concentrations below the IC_20_ (0.1 nM and 1.0 nM for IDC-1 and SUM-229PE, respectively) were used together with the corresponding IC_50_ of astemizole. In the box: C = Calcitriol, A = Astemizole. Results for Ki-67 and Eag1 gene expression in IDC-1 are shown in panels A and B, respectively. Ki-67 in SUM-229PE is shown in panel C. No significant regulation of Eag1 by astemizole alone or in combination was observed in SUM-229PE (data not shown). Results were normalized against GAPDH mRNA expression; vehicle values were set to one. N = at least 3, *P<0.05 vs control and each compound alone.

Considering that low calcitriol concentrations only when used concomitantly with astemizole significantly inhibited cell proliferation, we decided to investigate if this effect was also reflected at the gene level, in the expression pattern of Eag1 and Ki-67. Therefore, we tested two calcitriol concentrations below the IC_20_ (0.1 and 1.0 nM for IDC-1 and SUM-229PE, respectively) used together with the IC_50_ of astemizole. As depicted in figure 3A and B, calcitriol or astemizole alone slightly reduced Eag1 and Ki-67 gene expression in IDC-1; however, when used concomitantly, a significant inhibition of gene expression was observed for both genes. Similar results were obtained for Ki-67 in SUM-229PE (Fig. 3C). No significant regulatory effects of astemizole, alone or in combination with calcitriol, upon Eag1 gene expression were observed in SUM-229PE cells (data not shown).

**Figure 4:**
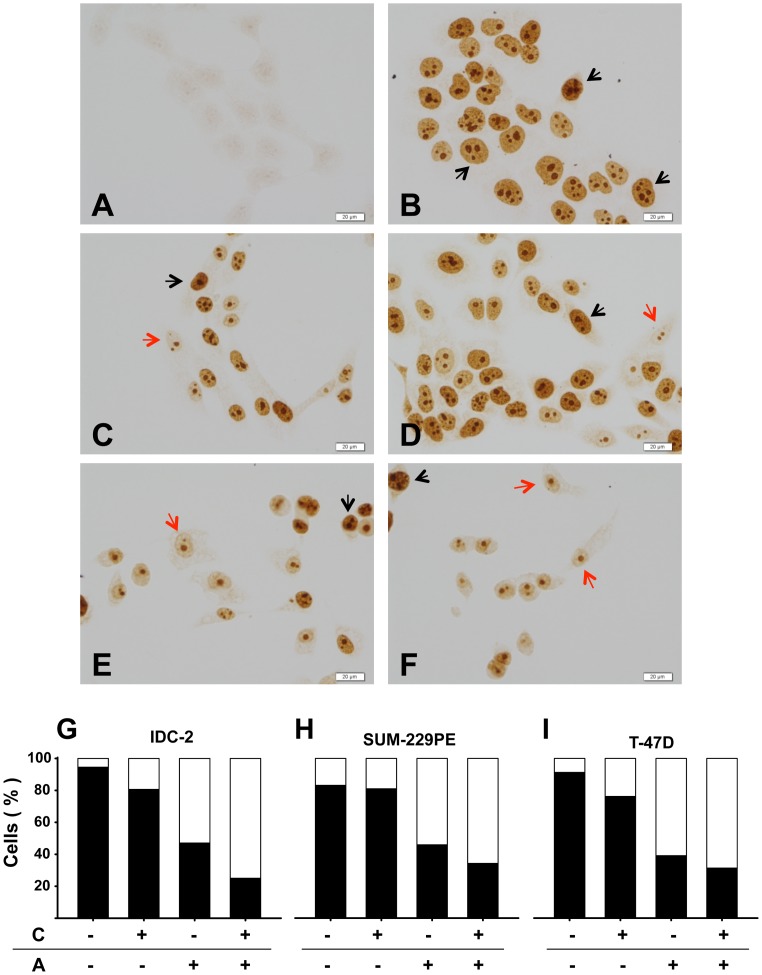
Immunocytochemistry of Ki-67 in breast cancer cells. Cells grown on glass coverslips were cultured in the presence of calcitriol, astemizole, both drugs (IC_20_ in all cases), or vehicle (-) during 48 h. In the box below the graphics: C = Calcitriol, A = Astemizole. No hematoxylin counterstaining was performed. Representative pictures of SUM-229PE are shown in panels A–F. Negative control in the absence of first antibody is shown in picture A. SUM-229PE cells were treated with: vehicle (picture B), astemizole (picture C), calcitriol (picture D), calcitriol + astemizole (pictures E and F). Black and red arrows indicate Ki-67-positive and negative cells, respectively. Similar results were obtained using T-47D and IDC-2 (data not shown). The percentage of Ki-67 positivity was calculated by counting total Ki67-positive nuclei ×100/total cell count. Three different cell lines were analyzed and results are shown in graphics as follows: IDC-2 (G), SUM-229PE (H) and T-47D (I). In all cases, percent of Ki-67-negative cells (white fraction) was higher compared to Ki-67-positive cells (black fraction) when coincubated in the presence of both compounds (G–I).

At the protein level, which was studied in IDC-2, SUM-229PE and T-47D, Ki-67 expression was consistently reduced by calcitriol and astemizole treatment, while concomitant incubations further reduced the percentage of positivity of this proliferation marker, as well as cell density, as depicted in the graphics and representative pictures shown in Figure 4. Interestingly, astemizole treatment also reduced cell size, which was more evident in SUM-229PE (Figure 4).

**Figure 5:**
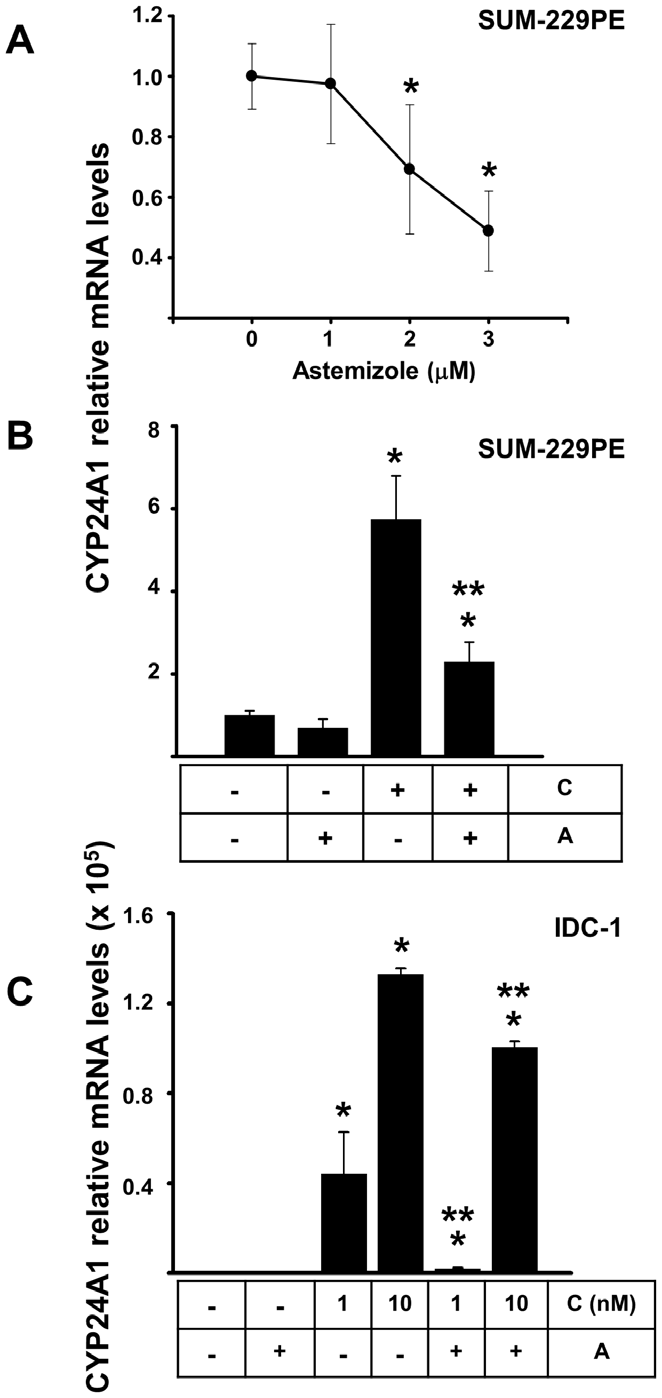
CYP24A1 transcriptional regulation by calcitriol and/or astemizole in breast cancer cells. Expression of CYP24A1 mRNA was studied in SUM-229PE (panels A and B) and IDC-1 panel C. Cells were incubated in the presence of increasing concentrations of astemizole (panel A) or with both drugs alone or in combination (panels B and C). In panels B and C astemizole was used at corresponding IC_50_ for each cell line. In panel B calcitriol was used at 0.1 nM. As depicted, astemizole downregulated basal CYP24A1 mRNA expression in SUM-229PE, whereas in both cell lines the coincubation of astemizole with calcitriol significantly reduced calcitriol induction of CYP24A1. Results were normalized against GAPDH mRNA expression and in all cases vehicle values were set to one. In the boxes below the graphics: C = Calcitriol, A = Astemizole. N = at least 3, *P<0.05 vs control, **P<0.05 vs each compound alone.

### Analysis of CYP24A1, VDR and CYP3A4 Regulation by Astemizole and Calcitriol

In order to better understand the molecular implications of calcitriol and astemizole co-treatment, we studied how astemizole affects CYP24A1 and VDR gene expression, as well as the regulation of CYP3A4 by calcitriol. Real time-PCR analysis showed higher basal CYP24A1 gene expression in T-47D, followed by SUM-229PE and finally the two cell lines derived from primary IDC tumors, where it was almost undetectable (data not shown). Considering the high basal expression of CYP24A1 in SUM-229PE, we used this cell line in order to study the effect of astemizole upon this gene. As shown in figure 5A, concentration-dependent inhibition of CYP24A1 mRNA expression was observed when cells were incubated in the presence of astemizole. As expected, addition of calcitriol significantly increased CYP24A1 mRNA expression in all cell lines studied (data not shown). However, coincubation of astemizole (IC_50_) with calcitriol (0.1 nM) significantly reduced calcitriol induction of CYP24A1 in SUM-229PE (Fig. 5B). In this cell line, astemizole was not able to abate CYP24A1 upregulation induced by calcitriol at higher concentrations. Similarly, in IDC-1, astemizole (IC_50_) also reduced calcitriol-dependent CYP24A1 mRNA upregulation, but, in contrast to SUM-229PE, this effect was observed at calcitriol concentrations of 1 and 10 nM (Fig. 5C).

**Figure 6:**
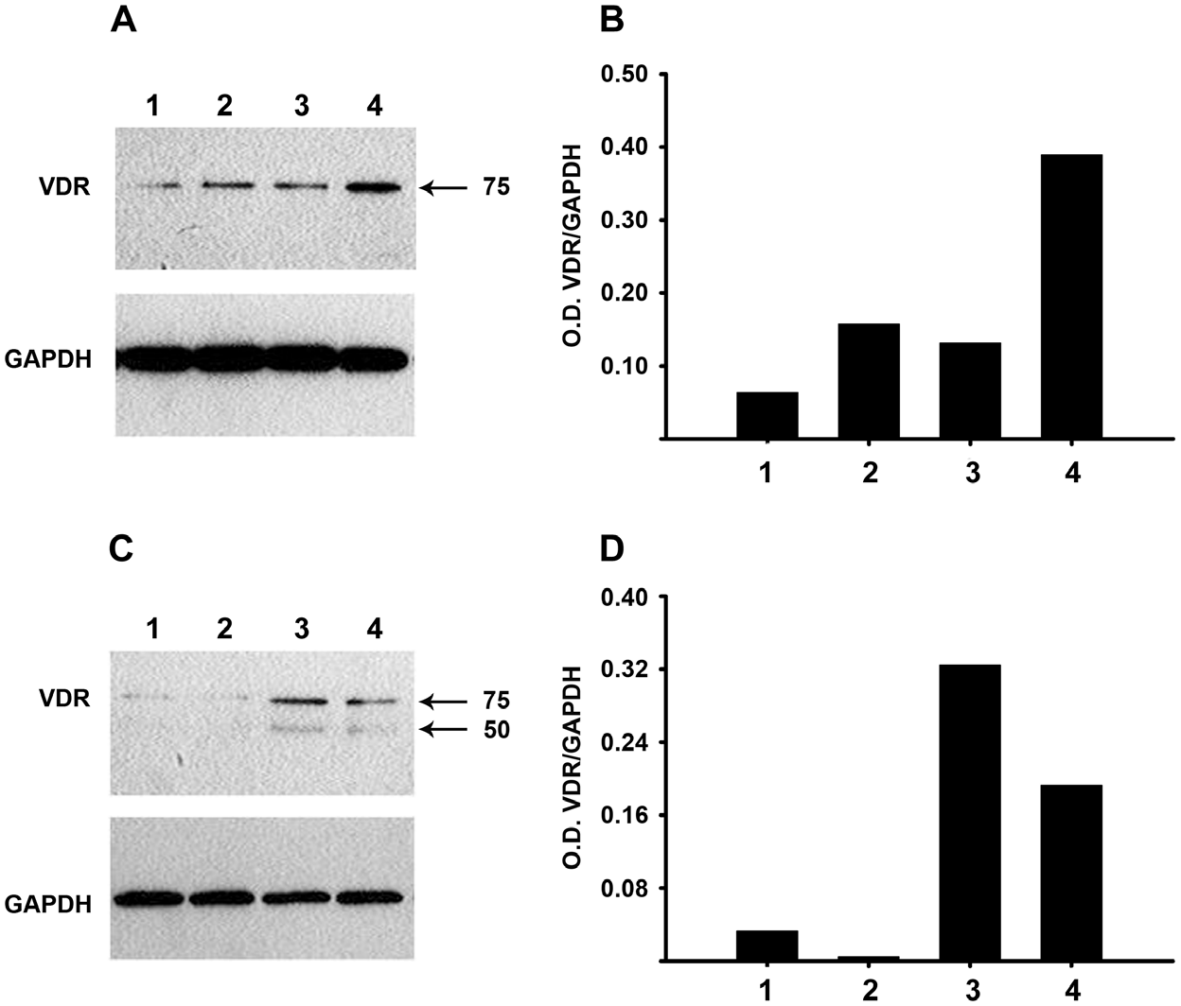
VDR protein is upregulated by astemizole. Western blot analysis of VDR in SUM-229PE (A and B) and T-47D (C and D). In panel A: Cells were incubated in the presence of vehicle (1), calcitriol (2, IC_20_), astemizole (3, IC_20_) or the combination of calcitriol + astemizole (4) during 48 h. In each case 50 µg protein were loaded per lane. In SUM229-PE, an immunoreactive protein of ∼75 kDa (indicated by an arrow) was slightly detected in vehicle-treated cells, whereas the treatment with calcitriol or astemizole improved its expression. The combined treatment of the cells in the presence of both drugs (4) further increased VDR expression, as observed after normalization against GAPDH optical density (O.D.), and depicted in panel B. Similar results were obtained using T-47D (C and D), but calcitriol alone did not induce VDR expression. However, the treatment with astemizole alone or in combination with calcitriol induced both the ∼75 kDa and the ∼50 kDa VDR species.

We then studied the regulation of VDR by astemizole. Results showed that astemizole stimulated VDR gene expression in both cell lines (1.92±0.32 and 2.28±1.08 folds over control for SUM-229PE and IDC-1; respectively, n = 3 and 5; respectively, *P*<0.05 vs. vh at 3 µM). Additionally, in SUM-229PE Western blots showed a slight immunoreactive VDR protein of ∼75 kDa in vehicle-treated cells, whereas the treatment with calcitriol or astemizole improved its expression. The combined treatment of the cells in the presence of both drugs further increased VDR expression, as depicted after normalization against GAPDH expression (Figure 6A and 6B). Similar results were obtained using T-47D, but calcitriol did not induce VDR expression. Nevertheless, the treatment with astemizole alone or in combination with calcitriol induced both the ∼75 kDa and the ∼51 kDa VDR species (Figure 6C and 6D).

**Figure 7:**
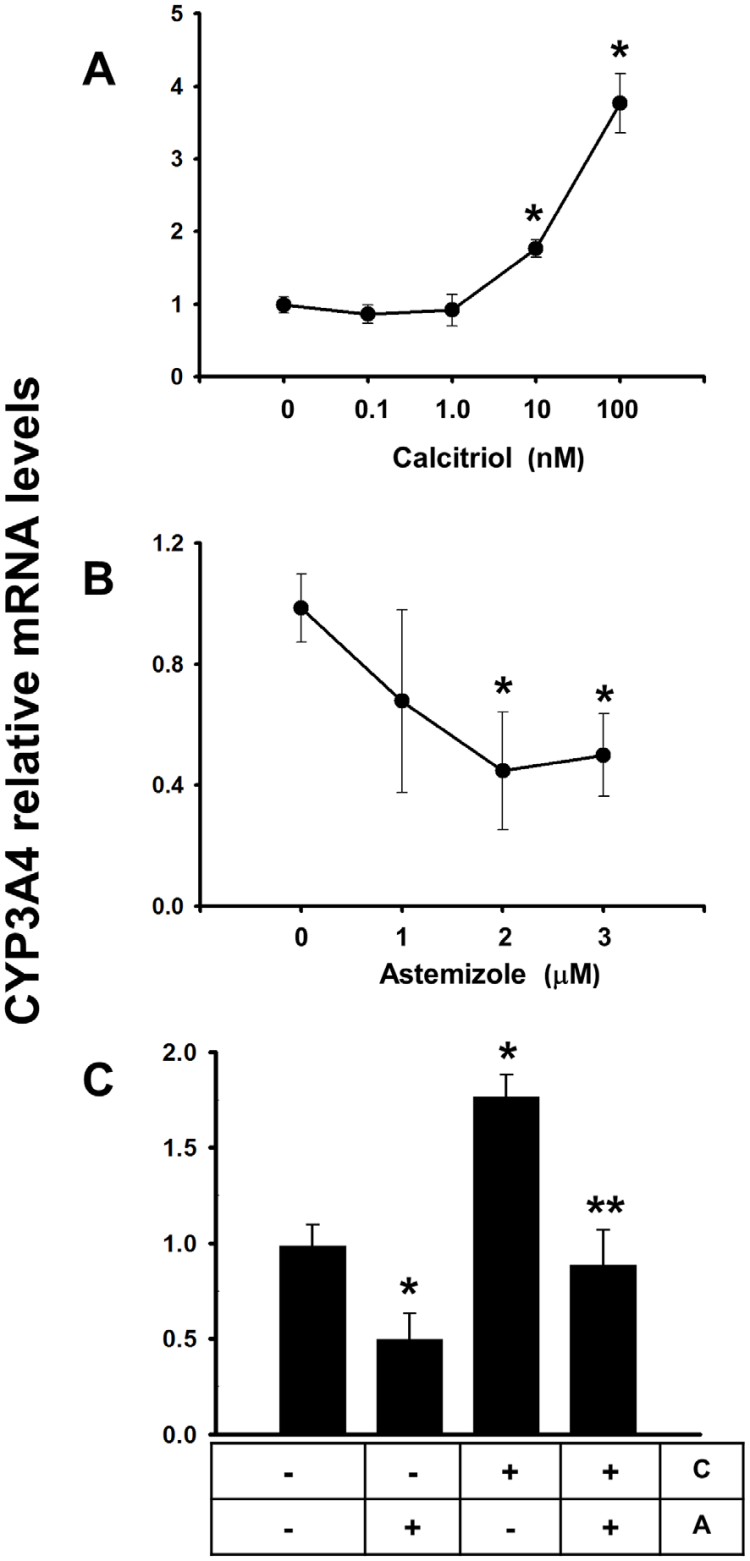
CYP3A4 gene expression regulation by calcitriol and astemizole in the hepatoma cell line HuH-7. Cells were incubated in the presence of increasing concentrations of calcitriol (panel A) or astemizole (panel B) during 24 hours. Afterwards RNA was extracted for real time PCR analysis. Combined effect of both astemizole (3 µM) and calcitriol (10 nM) is shown in panel C. Results were normalized against GAPDH mRNA expression and in all cases vehicle values were set to one. In the box below the graphic: C = Calcitriol, A = Astemizole. N = 3, *P<0.05 vs control. **P<0.05 vs each compound alone.

**Figure 8:**
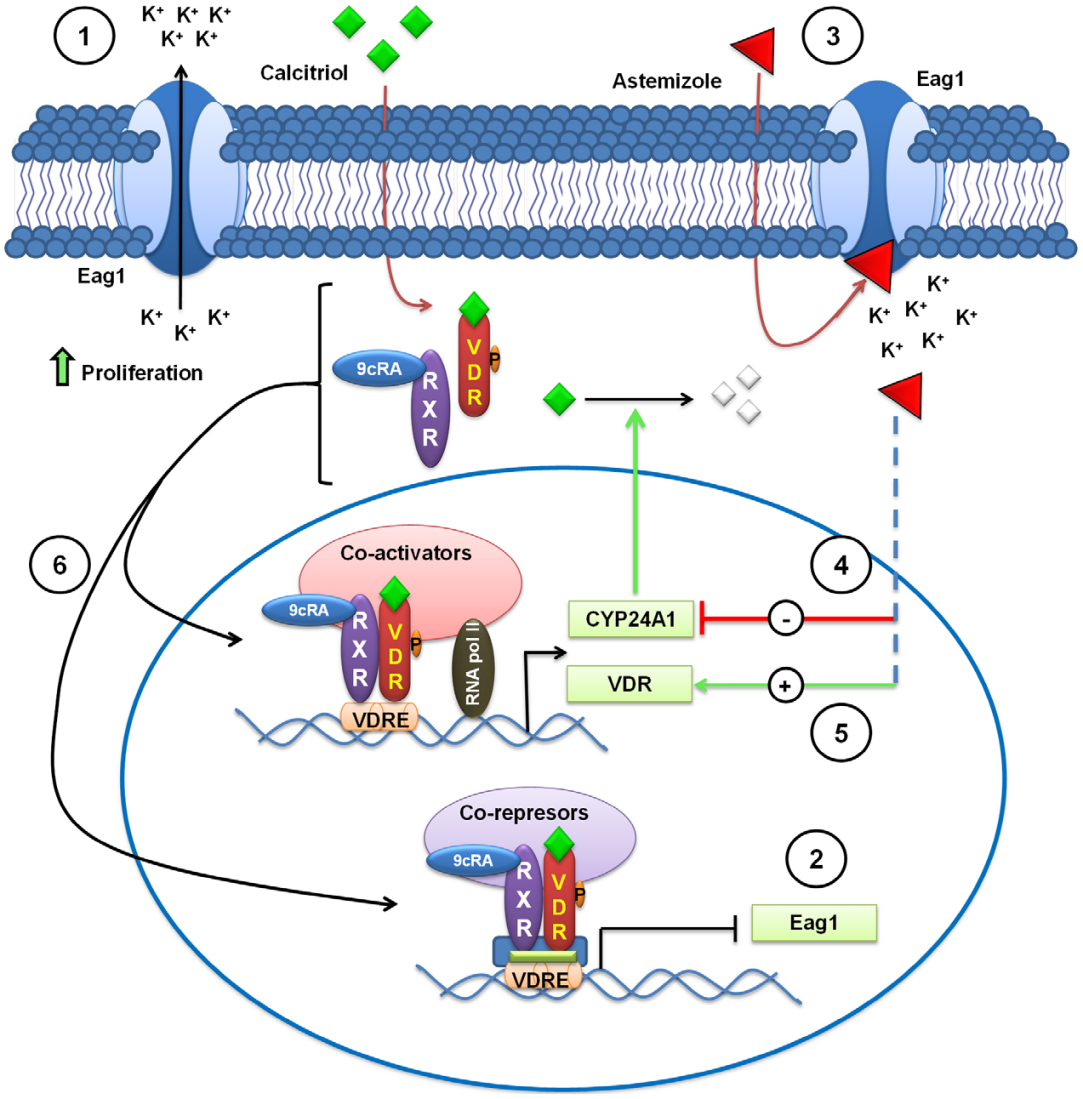
Caption to summary illustration. Astemizole synergizes calcitriol antiproliferative activity by downregulating CYP24A1, upregulating VDR and blocking Eag1 activity. Cell proliferation is stimulated by ion permeation through Eag1 channels (1). Eag1 gene expression and activity are blocked by calcitriol and astemizole, respectively (2 and 3). Astemizole downregulates CYP24A1 mRNA expression (4), allowing calcitriol to evade catabolism. Astemizole upregulates VDR expression (5) favoring calcitriol biological effects. Upon activation by calcitriol, the VDR forms heterodimers with the RXR and binds to vitamin D response elements (VDRE) on DNA resulting in expression or transrepression of specific gene products (6). VDR = vitamin D receptor; RXR = retinoid X receptor; 9cRA = 9-cis retinoic acid. CYP24A1 = cytochrome involved in calcitriol inactivation. Green rhomboid = calcitriol. Red triangle = astemizole. Dashed lines depict mechanistic events not yet fully clarified.

Considering that CYP3A4 is a calcitriol-responsive cytochrome involved in astemizole breakdown in the liver [27], we conducted experiments using a human liver cell line in order to gain insights into how calcitriol could affect astemizole metabolism *in vivo*. In HuH-7 cells, significant induction of CYP3A4 gene expression was achieved when incubating the cells in the presence of calcitriol (Fig. 7A). This effect was also observed in our cultured breast cancer cells, although to a lesser extent (data not shown). In contrast, astemizole inhibited basal CYP3A4 gene expression (Fig. 7B) and blocked calcitriol-dependent CYP3A4 induction (Fig. 7C), in a similar manner as that observed for CYP24A1 in breast cancer cells.

A schematic representation of the possible mechanistic interaction between calcitriol and astemizol in breast cancer cells is depicted in Figure 8.

## Discussion

To date, there is strong pre-clinical and clinical evidence to use the VDR as target for cancer therapy, since calcitriol has shown significant antineoplastic effects *in vivo* and *in vitro* [1], [28]. The initial concerns related to limitations in dose-escalation due to secondary hypercalcemia were left behind thanks to the studies of Beer, Muindi, Trump and colleagues, which showed that intermittent calcitriol dosing allowed substantial dose-escalation without dose-limiting toxicity [29]–[31]. Moreover, calcitriol antineoplastic effects are potentiated by many therapeutic agents including cytotoxic drugs, radiation, tamoxifen, glucocorticoids and ketoconazole, allowing for dose-reduction [1]. Calcitriol therapy seems promising especially for breast cancer, since malignant ductal epithelial cells generally express VDR, as shown in this and other studies [4], [10]. Taking these observations into consideration in addition to those showing Eag1, an oncogenic factor, as a target for calcitriol [4], [5], we aimed at investigating if astemizol, a blocker of Eag1 activity, increased the potency of calcitriol antiproliferative effects in breast cancer cells. A key aspect in targeted therapies is the accurate selection of those patients more likely to be benefited from drug treatment. Earlier reports have shown that high VDR and Eag1 expression is characteristic of breast tumors [10], [15]. Thus, tumors expressing both biomarkers should respond to calcitriol and astemizole in a similar manner as observed in the cells used in this study. VDR and Eag1 presence and bioactivity in breast cancer cells are; therefore, predictors to calcitriol and astemizole response, independently of estrogen or growth factor receptors status, as shown herein. Indeed, in this study, all cell lines studied were positive for VDR and Eag1. Accordingly, the concomitant treatment with calcitriol and astemizole synergistically reduced cell proliferation, independently of their molecular signature. This effect was also observed in T-47D cells, which were characterized by high CYP24A1 and low VDR mRNA expression. Our results also indicated that astemizole was able to potentiate the antiproliferative effects of calcitriol by a mechanism involving downregulation of CYP24A1 and upregulation of VDR expression, strongly suggesting that astemizole is able to improve calcitriol biological effects. Indeed, this observation is of relevance since clinically, the coadministration of calcitriol and CYP24A1-inactivating compounds can result in a significant increase in calcitriol plasma concentrations [32], with consequential therapeutic benefit in cancer patients, as has been previously described with ketoconazole [33]. Herein, we show for the first time that astemizole upregulated VDR expression. In SUM-229PE, incubations in the presence of calcitriol or astemizole stimulated, while concomitant addition of both drugs further induced VDR expression, corroborating qPCR analyses. In T-47D, calcitriol by itself did not induce VDR, which together with low and high basal VDR and CYP24A1 expression; respectively, might be the reasons why T-47D was rather resistant to calcitriol antiproliferative activity. However, the treatment of T-47D with astemizole alone or in combination with calcitriol induced two recognized VDR species, which explains why this cell line became so sensitive to the co-treatment. It is noteworthy to mention that previous studies from us and others have shown calcitriol-mediated effects through a high molecular weight protein that shares immunologic cross-reactivity with the 50-kDa VDR species [34], [35]. The participation of this VDR isoform in calcitriol antiproliferative effects deserves further investigation. While the regulation of VDR abundance is important for modulating calcitriol responsiveness in target cells, the mechanisms by which VDR levels are regulated are not so clear. Ligand-induced stabilization of the VDR protein is the main mechanism by which VDR levels are regulated [36], [37]; however, increased transcription may also participate, as shown in this and other studies [36], [38]. Our results in SUM-229PE suggest that calcitriol might be prolonging VDR protein lifetime, while astemizole may activate signaling pathways that induce VDR transcription. The sum of both mechanisms could explain VDR further increased expression after incubating the cells with the combination of both drugs. The astemizole-induced VDR upregulation may be physiologically relevant, as shown previously in models of prostate cancer where a slight increase in VDR synthesis significantly sensitized the cells to calcitriol antineoplastic effects without overt side effects [39].

An interesting observation in this study was that significant percent growth inhibition was achieved at the IC_20_ (or less) of each compound when used concomitantly, indicating that fewer drug is required to reach antiproliferative activity, which suggests that reduced dose-related toxicity could be feasible while retaining therapeutic efficacy in an *in vivo* combined treatment.

In addition, both astemizole and calcitriol negatively targeted Eag1 gene expression, which together with the functional blockade of Eag1 channel activity by astemizole, may further explain the highly effective antiproliferative effects of the combined treatment, as reflected herein in the synergistic inhibition of cell growth and Ki-67 expression. Indeed, concomitant incubation of cells with both compounds further inhibited the expression of the molecular proliferation marker Ki-67, which is frequently used as a pharmacodynamic indicator of therapeutic efficacy in breast cancer patients [40]. It is generally accepted that Ki-67 antigen staining increases during S phase and is further enhanced during G2 phase of the cell cycle; however, during the G1 phase Ki-67 expression decreases and is found as a weak staining [41]–[43]. In our study, we observed a consistent reduction in Ki-67 immunoreactivity after treating the cells with calcitriol, astemizole or their combination, suggesting that cells were arrested in G1 phase. Indeed, previous studies have shown that astemizole and calcitriol both induce arrest in G1 phase of the cell cycle [17], [44], [45].

Previously, we showed that calcitriol via VDR inhibited Eag1 gene expression reducing breast cancer cell proliferation [4] and Weber *et al* demonstrated that silencing Eag1 expression with siRNA resulted in similar antineoplastic effects [46]. Considering that Eag1 is also associated with malignant transformation and other hyperproliferative disorders such as cervical cancer [47], our results highlight Eag1 as a potential clinical oncogenic target as shown in this study with calcitriol in combination with astemizole. Since astemizole does not interact with the chromatin via nuclear receptors, but rather acts upon membrane Eag1 by selectively binding to open channels [48], its effects on gene expression modulation remain largely unknown. However, the fact that astemizole reduced Eag1 mRNA in this study might be secondary to H_1_ receptor antagonism and subsequent inactivation of downstream signaling pathways associated with generation of second messengers and proliferation. Alternatively, astemizole might be blocking Eag1 channels located in the inner nuclear membrane. These channels have been proposed to participate in nuclear K^+^ homeostasis or indirectly interact with the heterochromatin, both factors known to affect gene expression [49]. This issue clearly deserves further investigation. Nevertheless, it is noteworthy to mention that when SUM-229PE cells were treated with astemizole, they showed no change in Eag1 mRNA, yet they were growth inhibited fairly well by the treatment. Thus, the mechanism of astemizole growth inhibition is not likely via Eag1 downregulation, and might involve apoptosis, as suggested by the reduction in cell size observed in SUM-229PE, and studied elsewhere [50]. On the other hand, even if calcitriol consistently down-regulated Eag-1 in all cell lines tested, and the effect of both drugs combined upon inhibition of cell proliferation and Ki-67 expression was synergistic, the significant downregulation of Eag1 mRNA observed with the combination of astemizole plus calcitriol in IDC-1 was not detected in SUM-229PE cells. The explanation of this phenomenon might be related to the diversity among tumors; particularly, the heterogeneity in molecular signatures relevant to survival pathways of the specific cell line, and the cross-talk that takes place between signaling pathways. Indeed, differential responses to a combined treatment are to be expected, given the biological heterogeneity that prevails in cancer cells from different tumors, which is a feature also observed in patients under the same therapeutic regimen and that respond divergently. Inasmuch, the results in this study clearly show that the combined antiproliferative activity of calcitriol and astemizole is greater than the sum of the effects of each drug alone.

Calcitriol is an FDA-approved drug commonly indicated in the management of secondary hyperparathyroidism in patients with renal failure, whereas astemizole was withdrawn from U.S. and European markets in 1999 after concerns about the drug's safety. However, more than 30 countries still use it to treat either malaria or simple allergies.

Physiologic levels of calcitriol range from 0.05 to 0.16 nM; however, preclinical data indicate that significant growth inhibition requires calcitriol concentrations ≥ 1 nM [4]. On the other hand, clinical studies have demonstrated that under a weekly administration schedule, calcitriol may reach peak blood levels of 3–16 nM with little toxicity [21], [22]. In this study, mean IC_50_ of calcitriol for cell growth inhibition was 13.32 nM (not considering T-47D). However; when used in combination with astemizole, significant antiproliferative synergistic effects were reached even at the lowest calcitriol concentration tested (0.01 nM). Importantly, these effects were observed in ER negative cells using calcitriol at clinically achievable concentrations, which is to say with mean IC_20_ values of 1.82 nM (≈ 0.75 ng/mL). This concentration is considerably below to that observed in cancer patients treated safely with intravenous calcitriol (peak serum calcitriol = 6.68 ng/mL ≈ 16 nM) [21]. In the case of astemizole, reported therapeutic and toxic serum levels are 0.05 µg/mL (0.10 µM) and 14 µg/mL (30.5 µM), respectively [23]. Therefore, for anticancer applications the concentrations used in this study are within a compatible range (< 3 µM).

In addition, our results in HuH-7 cells showing a significant calcitriol-dependent induction of CYP3A4 gene expression, which encodes a liver enzyme involved in the metabolism of many drugs including astemizole, suggested that calcitriol might speed up astemizole metabolism. CYP3A4 upregulation by calcitriol has been shown also to occur in other cell types [51], and the presence of a functional VDRE in the promoter region of this gene supports our findings [52], [53]. However, no effects of calcitriol on CYP3A4 gene expression in the presence of astemizole were observed, strongly suggesting a compensatory mechanism between the stimulatory *vs* inhibitory effects of calcitriol and astemizole; respectively, on this gene. It is noteworthy to mention that CYP3A4 is also the major source of oxidative metabolism of calcitriol in the human liver [54]. In fact, in this study, calcitriol stimulated CYP3A4 expression both in the liver and breast cancer cells, suggesting a negative feedback control mechanism; which, interestingly, was dampened by astemizole. These results together with those showing the astemizole-dependant downregulation of CYP24A1 gene expression, involved in calcitriol inactivation, in breast cancer cells, provided further evidence to explain the increased antiproliferative activity of calcitriol in the presence of astemizole. Therefore, based on our results, in an *in vivo* context astemizole might potentiate calcitriol antineoplastic effects by inhibiting its degradation, while at the same time calcitriol may prevent astemizole side effects such as cardiac arrhythmias or *torsades de pointes* by stimulating CYP3A4 expression.

Altogether our data suggest that astemizole may modify the pharmacokinetics of calcitriol and implies the possibility of lowering the dose of this drug administered to cancer patients without sacrificing overall therapeutic efficacy. Alternatively, non-calcemic analogs of vitamin D could be taken into consideration for future studies in combination with astemizole. In this respect, endogenous VDR ligands such as 20-hydroxyvitamin D_2_ or synthetic vitamin D analogues designed to decouple antineoplastic activity from calcemic toxicity, are potentially attractive for novel therapies using vitamin D-derived drugs [55], [56].

In summary, this study established that calcitriol, when coincubated in the presence of astemizole, increased its antiproliferative activity in cells that expressed both the VDR and Eag1 genes. This effect was most probably due to astemizole-dependent VDR upregulation and CYP24A1 inactivation; as well as inhibition of Eag1 expression and activity mediated by both compounds (see summary illustration in Figure 8). Overall, results advice that less calcitriol dosing should be considered when administrating it combined with astemizole, since astemizole seems to improve calcitriol bioavailability and activity. Our data provide scientific bases for further pharmacodynamic and pharmacokinetic studies designed to test both compounds simultaneously in subjects affected with breast cancer, particularly those expressing VDR and Eag1. Moreover, the mechanistic description of the molecular interactions involved in the antineoplastic effects of astemizole and calcitriol defined herein suggests that the combined treatment could be beneficial to patients bearing solid or metastatic tumors with different molecular signatures, including those not sensitive to endocrine or immunological therapies.

### Conclusions

At clinically achievable concentrations, astemizole synergized calcitriol antiproliferative effects by downregulating CYP24A1, upregulating VDR and targeting Eag1. A clear evidence-based rationale is provided to test calcitriol in combination with astemizole as an adjuvant in the management of VDR/Eag1-expressing breast cancer tumors, regardless of the presence of other molecular markers such as ER, PR or Her2-neu.
